# The Effect of Self-Regulation on the Need for Psychological Help Through Happiness, Resilience, Problem Solving, Self-Efficacy, and Adjustment: A Parallel Mediation Study in Adolescent Groups

**DOI:** 10.3390/children12040445

**Published:** 2025-03-30

**Authors:** İhsan Akeren, Eyüp Çelik, İbrahim Erdoğan Yayla, Mustafa Özgöl

**Affiliations:** 1 Department of Psychological Counseling and Guidance, Faculty of Education, Bayburt University, Bayburt 69100, Turkey; ihsanakeren@bayburt.edu.tr (İ.A.); ibrahim.yayla2@ogr.sakarya.edu.tr (İ.E.Y.); 2 Department of Psychological Counseling and Guidance, Faculty of Education, Sakarya University, Sakarya 54050, Turkey; 3 Department of Psychological Counseling and Guidance, Faculty of Education, Ahi Evran University, Kırşehir 40100, Turkey; mustafa.ozgol@ahievran.edu.tr

**Keywords:** self-regulation, need for psychological help, parallel mediation, adolescents

## Abstract

**Introduction**: Adolescence is a developmental period in which the risk of mental problems is high. Failure to resolve the problems encountered during this period may result in the need for psychological help. Based on the literature review, this study aimed to examine self-regulation, which has the potential to reduce adolescents’ need for psychological help, and the mediation of happiness, resilience, problem solving, self-efficacy, and adjustment through this skill. **Methods**: In the cross-sectional correlational survey design study, 1013 adolescents (542 girls, 471 boys, mean age: 15.04 ± 1.75 years) aged 12–19 years and studying in middle and high school in Bayburt, Turkey were reached. Seven different questionnaires, valid and reliable in adolescent groups, were used to measure the study variables. **Results**: Correlation results showed that all predictors were positively correlated with each other and negatively correlated with the dependent variable, the need for psychological help. After testing the assumptions, the results of the parallel mediation analysis showed that happiness, problem solving, self-efficacy, and adjustment fully mediated the effect of self-regulation on the need for psychological help. Another finding is that resilience is not a predictor of psychological distress. **Conclusions**: The results are important because they provide insight for those working in the field of adolescent mental health in terms of understanding the factors through which self-regulation reduces the need for psychological help.

## 1. Introduction

Individuals face various challenges at many stages of their lives. One of them, adolescence, is a stormy period in which they experience rapid physical and cognitive changes, their relationships begin to deepen, and the likelihood of mental and social problems is high [1,2,3]. In particular, assuming that the process of identity formation and acquisition is an important developmental task in this period, it is suggested that obstacles and unmet needs that cannot be overcome in adolescence can cause traumatic scars, low self-perception, and difficulties in later life [4]. In fact, when adolescents are negatively affected by some difficulties they face and cannot overcome them, it becomes important for them to get help [5]. Therefore, it should be taken into consideration that adolescents may seek help in order to bring more effective solutions to difficulties and achieve better results.

Murray and McAdams [6] emphasized that seeking help is a basic need for individuals to maintain a healthy social life. In psychology, the concept of need refers to the absence of important conditions necessary for the cognitive, emotional, and social development of individuals [7]. Maslow [8] pointed out that the psychological needs he defined should be adequately met by the individual’s environment in order to prevent subjective discomfort and illness and even stated that these needs can be compared to basic elements such as vitamins, calcium, and salt that are necessary for the body. According to him, the lack of these needs makes the individual sick, their fulfillment heals, and their regular fulfillment prevents disease. In other words, it is accepted that the fulfillment of psychological needs is important for the mental health of individuals. On the contrary, when these needs are not met, disharmony and even psychopathological conditions can be observed. Based on this information, it can be said that it is inevitable for adolescents whose psychological needs are not met to feel the need for psychological help. The desire to meet needs leads individuals to act, and when these needs are not met in a healthy manner, it can lead to risks such as phone and social media addiction [9,10,11,12,13,14], mental disorders such as depression, eating disorders, non-suicidal self-harm, suicide attempts [15,16,17,18,19], and violent behavior [20]. In this regard, it can be said that meeting psychological needs has an important place in the prevention of adolescent psychopathology.

### 1.1. Self-Regulation and Need for Psychological Help

Given the emphasis on meeting psychological needs to reduce the need for psychological help [5], self-regulation skills emerge. Self-regulation is a multi-component set of skills that includes cognitive, emotional, and behavioral regulation, including skills such as delaying gratification and managing sudden impulses or reactions [21,22]. In short, self-regulation can be said to be an individual’s ability to manage themselves. Furthermore, Steinberg et al. [23] emphasize that self-regulation is the most important predictor of mental health and that developing this skill during adolescence is an important developmental task. Taken together, these findings suggest that individuals with higher self-regulation during adolescence may be better able to cope with the negative events they face.

There are many studies indicating that self-regulation and the need for psychological help are strongly correlated [24,25,26,27]. Examining these studies, it is found that individuals with high self-regulation skills can effectively manage their cognition, emotions, and behaviors, are aware of their psychological needs, and take an active role in meeting them. Therefore, we can conclude that they are healthier, more successful, happier, and less in need of psychological help.

### 1.2. Happiness

Studies on the relationship between self-regulation and happiness show that the two are generally positively correlated [28,29,30]. As mentioned above, self-regulation is the ability to control one’s emotions, thoughts, and behaviors in accordance with a specific goal. This skill is particularly important in stressful situations encountered in daily life and in achieving long-term goals [31,32]. Happiness, on the other hand, is the state of satisfaction with one’s life in general and the frequent experience of positive emotions [33,34].

When examining the relationship between happiness and the need for psychological help, higher happiness is associated with better mental health [35]. In this case, it can be observed that happy individuals need less psychological help because happy people generally experience fewer mental health problems such as stress, depression, and anxiety [11,36,37]. In general, high happiness may decrease the need for psychological help, while low happiness may increase this need. In fact, another study examining the relationship between self-regulation, happiness, and the need for psychological help [38] concluded that individuals with high self-regulation are less likely to seek psychological help because they are happier, which supports this assumption.

### 1.3. Resilience

Psychological resilience is an individual’s ability to survive and capacity for recovery in the face of challenging life events and conditions [39]. Similarly, psychological resilience is also defined as the ability to successfully adapt to life despite risky situations [40]. Considering the relationship between self-regulation skills and psychological resilience, it is known that self-regulation is an important skill in adapting to challenging life events and creating goals for the future [41]. Furthermore, the effective role of self-regulation in the development of psychological resilience stands out when considering skills such as the use of internal resources and impulse control in challenging life situations [42]. For this reason, self-regulation is reported to be one of the protective factors closely associated with psychological resilience, especially in vulnerable adolescents [43]. According to a similar result, self-regulation predicts psychological resilience, one of the intrinsic protective factors [44]. Moreover, there are studies that support the positive relationship between psychological resilience and self-regulation [29,45,46,47,48]. Therefore, it can be said that psychological resilience may be a mediating variable in the relationship between self-regulation and the need for psychological help. This is because it has been reported that individuals who are psychologically healthy have less need for psychological help because they are more competent in coping with difficulties [49,50,51].

### 1.4. Problem Solving

Problem solving is the ability of individuals to effectively identify, analyze, and overcome the challenges they face [52]. Self-regulation plays a critical role in each stage of the problem-solving process. Self-regulation enables individuals to focus on the process and effectively maintain their behavior during the stages of defining the problem, planning and implementing a solution strategy, and evaluating the results [53,54]. Problem solving skills increase an individual’s ability to cope with daily stressors and life events. However, inadequate problem-solving skills may make it difficult for individuals to cope with stress and thus increase psychological help-seeking behavior [55,56,57,58]. From this perspective, it can be said that individuals who are successful in problem solving generally tend to solve their problems on their own and therefore have a lower need for psychological help.

### 1.5. Self-Efficacy

The relationship between self-efficacy, another variable examined in the study, and self-regulation, that is, how individuals’ beliefs about their own abilities and the abilities they use to achieve their goals interact, is an important issue. While self-efficacy is the belief in the ability to perform a task or achieve a goal, self-regulation is the ability to manage oneself in achieving goals [59,60]. Studies have reported that self-regulated individuals generally have high self-efficacy and use self-regulation more actively to achieve their goals. In summary, self-regulation increases an individual’s sense of control and confidence in their own performance [61,62,63].

Individuals with high self-efficacy perceptions will feel less need for psychological help because they have characteristics such as positive self-evaluation, believing that they have the power to overcome problems, clearly identifying their needs, and not perceiving seeking help as a weakness. There are many studies that support the idea that self-efficacy and the need for psychological help are related [5,64,65,66]. In summary, it can be concluded that individuals with high self-efficacy tend to solve problems on their own and therefore have a low need for psychological help.

### 1.6. Psychological Adjustment

Psychological adjustment and self-regulation are two critical concepts that have a significant impact on an individual’s mental health and functioning. Psychological adjustment is defined as an individual’s ability to cope with stress, change, and difficulties and to remain psychologically balanced [67]. Self-regulation is the ability to control, manage, and direct one’s emotions, thoughts, and behaviors in order to achieve one’s goals [68].

Individuals with developed self-regulation skills tend to be more flexible and resilient in the face of stressful situations, which increases their psychological adjustment. Research shows that individuals with high self-regulation skills can effectively cope with stress and depression, and their psychological flexibility increases [69]. Self-regulation is the ability to control one’s emotional reactions. Individuals with high ability to manage their emotions are more resistant to negative emotional experiences and this enables them to maintain their psychological adjustment. Gross [70] stated that emotion regulation strategies have a significant impact on an individual’s psychological well-being. Self-regulation skills help individuals achieve their long-term goals and maintain their psychological adjustment in the face of difficulties. Individuals with high self-regulation skills tend to be more resilient in the face of disappointment and failure [71]. As a result, it is believed that there is a reciprocal relationship between psychological adjustment and self-regulation, and that both play a critical role in determining overall psychological health. Those who are successful at self-regulation generally have higher levels of psychological adjustment [72]. It can be assumed that psychologically adaptive individuals have high emotional awareness, self-acceptance, flexibility, positive thinking, can cope with stress, are realistic, and have a sense of responsibility, and therefore play a more effective role in meeting their psychological needs. Indeed, there are studies that support our assumption that adjustment is effective in addressing the need for psychological help [73,74,75].

### 1.7. Theoretical Perspectives

**Figure 1:**
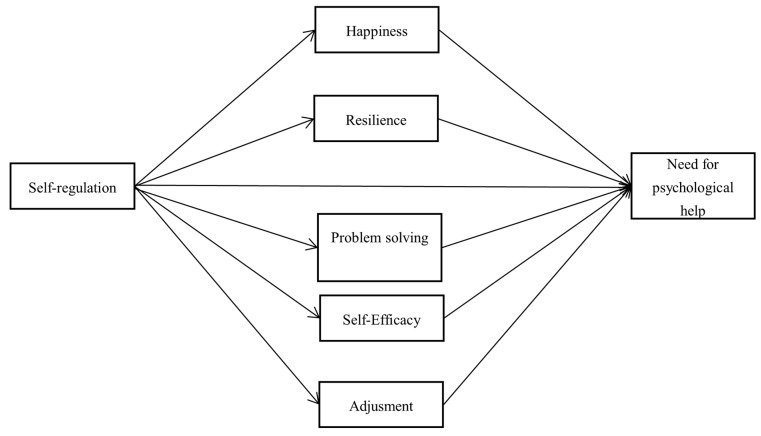
Hypothetical model of the effect of self-regulation on the need for psychological help.

To the best of our knowledge, the relationship between self-regulation and the need for psychological help has been proven in many studies [5,24,26]. However, there are no studies on the variables through which self-regulation predicts the need for psychological help. Based on our literature review, we conclude that self-regulation strengthens psychological resilience, happiness, problem solving, self-efficacy, and psychological adjustment, while reducing the need for psychological help. Taking this finding one step further, the possibility that related factors mediate the effect of self-regulation on the need for psychological help comes to mind. From this point of view, the aim of the study is to test the mediation of the variables mentioned in the effect of self-regulation on the need for psychological help. In this way, this effect will be expanded and better understood. In addition, by determining the extent to which the study variables are effective on the need for psychological help, it will also provide an idea to the field workers who aim to improve the mental health of adolescents. In this sense, we have listed the hypotheses of our study as follows and presented the hypothetical model in Figure 1.

**H1.**  *If self-regulation increases in adolescents, then the need for psychological help decreases.*

**H2.**  *If adolescents’ happiness increases, the relationship between self-regulation and the need for psychological help weakens.*

**H3.**  *If adolescents’ resilience increases, the relationship between self-regulation and the need for psychological help weakens.*

**H4.**  *If adolescents’ problem-solving skills increase, the relationship between self-regulation and the need for psychological help weakens.*

**H5.**  *If adolescents’ self-efficacy increases, the relationship between self-regulation and the need for psychological help weakens.*

**H6.**  *If adolescents’ psychological adjustment scores increase, the relationship between self-regulation and the need for psychological help weakens.*

**H7.**  *If adolescents’ happiness, resilience, problem solving, self-efficacy, and psychological adjustment increase, the relationship between self-regulation and the need for psychological help weakens.*

## 2. Materials and Methods

### 2.1. Participants

The participants of the current study, which had a cross-sectional correlational survey design, consisted of 1013 adolescents (542 girls, 471 boys, mean age: 15.04 ± 1.75, range: 12–19) studying in the 7th, 8th, 9th, 10th, 11th, and 12th grades of eight middle schools and six high schools in Bayburt, Turkey. A priori calculation using G*Power (version 3.1.9.7) suggested a total sample size of 424 (test family: F, statistic: linear multiple regression, effect size: 0.05, α error prob: 0.05, power (1 − β error prob): 0.95, number of predictors: 6). A two-stage sampling procedure was used to identify schools and reach participants. In the first stage, a list of all middle and high schools in the city center was generated using cluster sampling. Then, simple random sampling was used to determine the clusters (middle and high schools) to be included. Convenience sampling was used to reach the students in the schools, taking into account the intensity of the courses, the permission of the teachers, and the volunteerism of the students. Data were collected with as equal a representation as possible of gender and grade level.

### 2.2. Instruments

**Self-regulation (Self-Regulation Scale for Adolescents-SRS):** Developed by Kaşıkcı and Öğülmüş [76] to measure adolescents’ general self-regulation skills, the scale consists of 11 items and a single dimension (e.g., “I can keep in mind information that will be useful in reaching my goals”) and is scored on a scale of 1 to 5 (not at all suitable for me–completely suitable for me). The scores that can be obtained from the scale, which has no reverse-scored items, vary between 11 and 55, with higher scores being interpreted as higher self-regulation skills. The instrument, which was determined to explain 51% of the total variance in the development study and whose internal consistency coefficient was reported as 0.90, was also reliable in the current sample (Cronbach’s alpha = 0.76).

**Happiness (Oxford Happiness Questionnaire Short Form-OHQ):** The scale developed by Argyle et al. [77] to measure happiness in university students was later converted into a unidimensional short form [78]. In the development study, the 8-item structure, with responses ranging from 1 to 6, was adapted to Turkish culture [79] in a 7-item form. In addition, the responses to the questions (e.g., “I am satisfied with everything in my life”) are rated on a scale of 1 to 5 (strongly disagree to strongly agree) to ensure Turkish meaning and comprehensibility. The scores that can be obtained from the scale, in which two items are reverse scored, vary between 7 and 35, with higher scores interpreted as more happiness. The instrument, which was determined to explain 64.3% and 39.74% of the total variance in the development and adaptation studies, respectively, and whose internal consistency coefficient was reported as 0.90 and 0.74, was also reliable in the current sample (Cronbach’s alpha = 0.73).

**Psychological resilience (The Brief Resilience Scale-BRS):** Developed by Smith et al. [80] to assess the ability of undergraduates and individuals with various medical illnesses to recover from stress (or recovery) and thus test a resilience scale, the instrument has a unidimensional 6-item form. Questions (e.g., “It is hard for me to snap back when something bad happens”) are answered on a scale of 1 to 5 (strongly disagree–strongly agree). The lowest and highest scores that can be obtained from the scale, in which three items are reverse scored, are in the range of 6–30. The internal consistency of the scale, which explained 51% to 67% of the total variance in the developmental study, ranged from 0.80–0.91. The scale, which explained 54.66% of the total variance in the adaptation study and whose internal consistency coefficient was reported as 0.83 [81], is also reliable for the current sample (Cronbach’s alpha = 0.79).

**Problem solving (Coping Scale for Children and Youth-CSCY):** Developed by Brodzinsky et al. [82] to determine the coping behaviors of children and adolescents, it has a 29-item form with four dimensions: assistance seeking, cognitive-behavioral problem solving, cognitive avoidance, and behavioral avoidance. In the adaptation study for Turkish culture, the instrument, which retained the factor structure of the original study, was reduced to 24 items [83]. Questions (e.g., “I took a risk and tried a new way to solve the problem”) are answered on a scale of 1 to 4 (never–sometimes–often–very often). In the current study, in which 7 questions belonging to the cognitive-behavioral problem-solving dimension was used, the lowest and highest scores that can be obtained vary between 7–28, and higher scores are interpreted as increased problem-solving skills. The measurement tool, which was reported to explain 44% and 40% of the total variance in the development and adaptation studies, respectively, and whose internal consistency coefficient was reported as 0.81 and 0.79, respectively, is also reliable for the current sample (Cronbach’s alpha = 0.79). CFA results in our study indicated that the problem-solving subscale was valid in adolescent groups (χ2/df: 1.99, GFI: 0.99, CFI: 0.99, RMR: 0.01, RMSEA: 0.03).

**Self-efficacy (Generalized Self-Efficacy Scale-GES):** The 20-item instrument first developed by Jerusalem and Schwarzer in 1981 to assess individuals’ self-efficacy was reduced to a 10-item unidimensional version by the same researchers [84]. The Turkish cultural adaptation study was conducted with a sample of undergraduate and graduate students [85]. Questions (e.g., “It is easy for me to stick to my aims and accomplish my goals”) are answered on a scale of 1 to 4 (not at all true–exactly true). The lowest and highest scores that can be obtained from the scale are between 10 and 40, and no items are reverse-scored. The internal consistency of the scale is reported to be 0.86 in studies using versions of it in 25 languages. The instrument, which was found to explain 47% of the total variance in the adaptation study and whose internal consistency coefficient was calculated as 0.83, is also reliable for the current sample (Cronbach’s alpha = 0.82). CFA results in our study indicated that the scale was valid for measuring adolescent self-efficacy (χ2/df: 2.69, GFI: 0.98, CFI: 0.97, RMR: 0.02, RMSEA: 0.04).

**Psychological adjustment (Brief Psychological Adjustment Scale-BASE-6):** Developed to assess individuals’ general psychological adjustment, this scale has a unidimensional 6-item form [86]. Questions (e.g., “How much has emotional distress interfered with your ability to perform at work, school, etc. this week?”) are answered between 1 and 7 (not at all–extremely). Adaptation to Turkish culture was conducted on a sample of undergraduate students [87]. The possible scores range from 6 to 42, and higher scores are interpreted as a decrease in psychological adjustment. In our study, where psychological adjustment was considered as a positive variable, all responses were reverse coded and the total score was calculated. The internal consistency coefficient, reported as 0.87–0.93 in the development study, was calculated as 0.88 in the adjustment study and 0.87 in the current study. The CFA results in our study indicated that the instrument was valid for measuring adolescents’ psychological adjustment (χ2/df: 1.77, GFI: 0.99, CFI: 0.99, RMR: 0.05, RMSEA: 0.03).

**Need for psychological help (Psychological Help Need Scale Adolescent Form-PHNS):** Developed by Ay [5], this scale determines the level of satisfaction of the needs for security, belonging and love, esteem, self-actualization, other than the physiological needs identified by Maslow, which, if not satisfied, would make the individual sick [8,88]. The instrument adapted for adolescent groups maintained its four-factor structure and was reduced to 17 items [89]. The questions (e.g., “I am someone who is called and asked enough by my loved ones”) are answered between 1 and 5 (I don’t disagree at all–I completely agree). The minimum and maximum scores are between 17 and 85, and higher scores are interpreted as an increased need for psychological help. The internal consistency of the scale, which was found to explain 51.11% and 53.98% of the total variance in the development and adaptation studies for adolescent groups, respectively, was reported as 0.91 and 0.86. The instrument was also found to be reliable in the current sample (Cronbach’s alpha = 0.90).

### 2.3. Procedure

After getting the necessary permissions from the authorities, the researchers visited each school one by one in May–June 2024. After introducing themselves to the participants and explaining the purpose of the study and how to answer the questions, they delivered the questionnaire forms to those who volunteered to participate, and asked them to answer the questions on the condition that their parents signed the Informed Parental Consent Form since they were under 18 years of age. They were informed that no incentive would be given to them for this process. The forms completed under these conditions were collected the next day by visiting the schools again. The inclusion criteria were their own voluntariness and consent, as well as the signed consent of their parents for those under the age of 18, not having any mental disorder, not having a previous psychiatric diagnosis, and not being on ongoing treatment with medication. The answer sheets of students who did not volunteer to participate, who did not have the signed consent of their parents even if they volunteered, and who had psychiatric diagnoses were excluded.

### 2.4. Data Analysis

Of the 1036 completed questionnaires, six were excluded from the dataset because they did not include gender, year and age. Missing data analysis determined that there were 12 (1.2%) missing data in PHNS question 9, and 10 (1%) missing data in GES question 6 of the 64-question questionnaire form, while the missing rate in responses to other questions was less than 1%. The mean of the series method was used to replace missing values. To ensure normality, 17 of the standardized total scores other than ±3.29 were excluded from the data set. The histograms of each score were then examined, and finally skewness and kurtosis were calculated for the remaining 1013 data. As a prerequisite, mediation assumptions were also tested. Confirmatory factor analysis (CFA) and Cronbach’s alpha were used to test the validity and reliability of the measures, some of which had not been used in adolescent groups, in the current sample. Correlations were used to determine the relationships between variables, and the Hayes’ Process macro 4.2 plug-in (Model 4) integrated into SPSS 27 was used for parallel mediation analysis.

## 3. Results

### 3.1. Testing Mediation Assumptions

Hayes [90] states that some assumptions must be met in order to conduct a mediation analysis and lists them as normality, linearity, homoscedasticity, and independence. In testing normality, as previously mentioned, responses outside ±3.29 of the scale total scores, whose z-scores were calculated based on Tabachnick and Fidell’s [91] criteria, were excluded. The P-P plot created to test linearity showed that the residuals were densely clustered on the diagonal line, so there were no curves indicating a violation of linearity. The scatterplot generated to test for homoscedasticity shows that the points are scattered and there is no data to indicate heteroscedasticity. The Durbin–Watson value used to test for independence was calculated to be 1.953. Considering that according to Field [92] this value should be between 1 and 3, especially close to 2, it can be said that the errors are independent. Finally, the fact that the tolerance values calculated to test collinearity are greater than 0.10 (0.477 to 0.648) and the VIF values are less than 10 (1.550 to 2.098) can be interpreted as there is no multicollinearity problem among the predictors [93].

### 3.2. Descriptive Statistics and Correlations

Table 1 presents the distribution and correlation results of the participants’ mean scores on the measures and some demographic information. Pearson’s correlation was used because the skewness and kurtosis values of the total scores and chronological age were ±1.5, and Spearman’s correlation was used because the grade level was ordinal [91]. The results showed that SRS, OHQ, BRS, CSCY, GES, and BASE-6 were positively correlated with each other and all were negatively correlated with PHNS (*p* < 0.001). However, when analyzing Table 1, chronological age was negatively associated with OHQ, BRS, GES, and BASE-6, but not with SRS, CSCY, and PHNS. The relationship between participants’ grade level and the variables assessed was similar to that of chronological age. In addition, unlike chronological age, grade level was found to be positively associated with PHNS at a low level.

### 3.3. Mediation Results

**Figure 2:**
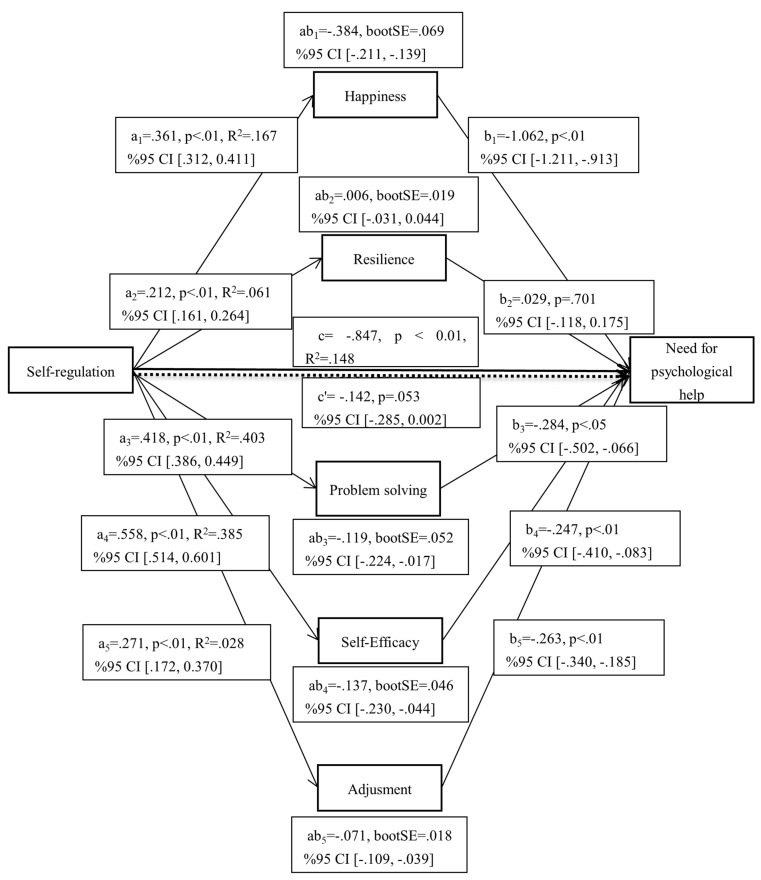
Parallel mediation results. a_1_, a_2_, a_3_, a_4_, a_5_: Effect of self-regulation on mediators; b_1_, b_2_, b_3_, b_4_, b_5_: Effect of mediators on the need for psychological help; ab_1_, ab_2_, ab_3_, ab_4_, ab_5_: Effect of self-regulation on the need for psychological help through mediators.

To determine whether happiness, resilience, problem solving, self-efficacy, and adjustment have mediating roles in the effect of self-regulation on psychological distress, regression analysis was used based on the bootstrap method (5000 bootstraps) and 95% confidence intervals [90]. The significance of indirect effects was determined by whether or not the confidence intervals included zero. The results are shown in Figure 2.

Figure 2 shows that the total effect of self-regulation on the need for psychological help is significant (c = −0.847, *p* < 0.01, R^2^ = 0.148, F_(1, 1011)_ = 176.081), but its direct effect is not significant in the model including mediators (c′ = −0.142, *p* > 0.05, se = 0.073, t = −1.936). The mediators of the independent variable self-regulation were happiness (a_1_ = 0.361, *p* < 0.01, R^2^ = 0.167, F_(1, 1011)_ = 203.264), psychological resilience (a_2_ = 0.212, *p* < 0.01, R^2^ = 0.061, F_(1, 1011)_ = 65.506), problem solving (a_3_ = 0.418, *p* < 0.01, R^2^ = 0.403, F_(1, 1011)_ = 683.834), self-efficacy (a_4_ = 0.558, *p* < 0.01, R^2^ = 0.385, F_(1, 1011)_ = 632.694), and psychological adjustment (a_5_ = 0.271, *p* < 0.01, R^2^ = 0.028, F_(1, 1011)_ = 28.640). When we look at the mediating variables, we see that happiness (b_1_ = −1.062, *p* < 0.01, se = 0.076, t = −13.979), problem solving (b_3_ = −0.284, *p* < 0.05, se = 0.111, t = −2.561), self-efficacy (b_4_ = −0. 247, *p* < 0.01, se = 0.083, t = −2.961), and psychological adjustment (b_5_ = −0.263, *p* < 0.01, se = 0.039, t = −6.665) had significant effects on the need for psychological help. Psychological resilience did not have a significant effect (b_2_ = 0.029, *p* > 0.05, se = 0.075, t = 0.384).

In the tested model, there are five indirect effects examined in order to reveal the mediation results. While the mediation effect is not significant in one of them, it is significant in the other four. In the effect of self-regulation on the need for psychological help, happiness (ab_1_ = −0.384, se = 0.069, 95% CI [−0.211, −0.139]), problem solving (ab_3_ = −0.119, se = 0.052, 95% CI [−0.224, −0.017]), self-efficacy (ab_4_ = −0. 137, se = 0.046, 95% CI [−0.230, −0.044]), and psychological adjustment (ab_5_ = −0.071, se = 0.018, 95% CI [−0.109, −0.039]), while the mediating roles of psychological resilience were significant (ab_2_= 0.006, se = 0.019, 95% CI [−0.031, 0.044]). The completely standardized indirect effects (K^2^) in mediation were calculated as −0.175 for happiness, −0.054 for problem solving, −0.063 for self-efficacy, and −0.032 for psychological adjustment. When calculating the effect size, if K^2^= 0.01, it is interpreted as a low effect, if it is close to 0.09, it is interpreted as a medium effect, and when it is close to 0.25, it is interpreted as a high effect [94]. In this context, it can be said that the first indirect effect of the current study is high, the third and fourth indirect effects are medium, and the fifth indirect effect is low.

In simpler terms, self-regulation positively predicts all mediators, and all of them, except resilience, reduce the need for psychological help. In addition, self-regulation reduces the need for psychological help through mediators other than resilience. When the model is evaluated as a whole, self-regulation has no direct effect on the need for psychological help.

## 4. Discussion

This study examined the mediation of some psychological factors identified in the literature review on the effect of adolescents’ self-regulation skills on their need for psychological help. It was concluded that happiness, psychological adjustment, problem solving, and self-efficacy mediated this effect, i.e., these variables reduced the need for psychological help, and happiness had the strongest mediating role among them. However, the same cannot be said for psychological resilience. As chronological age, which is one of the demographic factors, and grade level, which is almost perfectly positively correlated with it, increase, the means of OHQ, BRS, GES, and BASE-6 decrease, albeit to a limited extent, and the need for psychological help increases with grade level. This result is in line with the reports of previous studies [14,95]. Considering that these relationships are very weak, it can be said that the risks related to mental health in adolescents increase with age and, of course, with grade level, albeit partially.

The need for psychological help resulting from unmet psychological needs is an important concept in adolescent mental health. This is because recent studies have shown that unsatisfied psychological needs have negative consequences and risks such as addiction [96,97,98,99]. When reviewing the literature, it is stated that self-regulation is a strong predictor of the need for psychological help [24]. However, no study was found that examined which variables mediate this relationship. No study has been found that has extended the effect of self-regulation, which is subjective, closer to the self, has a predominant cognitive aspect, and therefore has an abstract nature, on the need for psychological help, which is characterized by emotionality. We believe that this situation limits a better understanding of the relationship between the two, which is why we can say that the planned study is unique in this respect.

The strongest mediation effect in this study is seen in the happiness variable. In other words, happiness has the lion’s share in reducing the need for psychological help. Since it is known that individuals with high self-regulation skills are competent in controlling their own emotions, thoughts, and behaviors, it is common for them to be happy in their lives [100]. It is reported that happy individuals have a high awareness of their psychological needs and are more skillful in satisfying them [101]. It is also known that those with self-regulation skills experience a positive effect more often and feel less need for psychological help [26,37]. In general, we can interpret that self-regulated behaviors increase happiness, and with increased happiness, there is less need for psychological help.

If we consider problem solving, which is another variable included in the equation as a mediator in this study that examines the relationship between self-regulation and the need for psychological help, Shoaakazemi et al. [102] stated that individuals with high self-regulation skills like to engage in metacognitive activities, try to master the problem instead of escape behavior when faced with problems, and have self-confidence and healthy evaluations of themselves. In addition to this information, it has been stated that individuals with high problem-solving skills are able to cope with life difficulties, have strong social networks and high life motivation, and therefore feel a lower need for psychological help [56]. Interpreting the findings on the mediation of problem solving in general terms, it can be said that self-regulation provides important resources for this skill, that those who can solve problems are more successful in coping with difficulties, and that problem solving supported by self-regulation reduces the need for psychological help.

The finding that self-efficacy, another mediating variable, is an important factor in reducing the need for psychological help is also supported by the literature [64]. In other words, self-regulation skills reduce the need for psychological help through self-efficacy. The adaptive skills provided by self-regulation, self-awareness, and self-confidence gained through self-efficacy play an effective role in perceiving and addressing the psychological needs of individuals in their current position.

When the mediating effect of psychological adjustment was examined, it was seen that it increased with self-regulation and thus reduced the need for psychological help. It has been reported that individuals with high psychological adjustment can cope with stressful situations more effectively and perceive higher levels of social support [74]. Given that individuals with low psychological adjustment are prone to have difficulty adapting to change, and have low motivation and life satisfaction, it is inevitable that they will seek psychological help [103,104,105].

Although the mediation of happiness, problem solving, self-efficacy, and psychological adjustment in the relationship between self-regulation and psychological help examined in the current study is consistent with the findings of previous studies, we cannot say the same for resilience. Contrary to studies reporting that psychological resilience reduces the need for psychological help [51,106], the lack of such an effect in our study was obviously a surprising finding for us. We should add, however, that the predictive relationships we examined are not only resilience and the need for psychological help. We believe that the nature of the structural equation model (SEM) will help us to understand this result. SEM is an advanced statistical method in which multiple variables are included in the equation and the complicated causal relationships between them are examined simultaneously [107]. In this sense, we examined the effect of a total of six variables on the need for psychological help and found that resilience was not effective. If we had ignored the others and focused only on the effect of resilience, the linear regression results based on the current data set would have indicated that it reduced the need for psychological help by approximately 13%, but this time the conclusion we reached from a narrow perspective would have been a mistake. In summary, the fact that resilience is not effective in reducing the need for psychological help in the presence of other factors in the current study is explained by our multivariate analysis technique (SEM).

In our current study, when happiness, self-efficacy, problem solving, and adjustment are considered together, it is possible to reach an important conclusion that self-regulation does not have a direct significant effect on the need for psychological help, but it is not possible to say which of these variables is a specific mediator. In terms of variables, happiness, self-efficacy, problem solving, and adjustment each play a role in this effect. According to Hayes [90], mediation is the explanation of part of the effect of the independent variable (X) on the dependent variable (Y) by another variable (M) between the two. If M cannot fully explain the observed relationship between X and Y, it is partial mediation, whereas if M is fully explained by an indirect mechanism, it is full mediation. These explanations caution, however, that full mediation is no more valuable or desirable than partial mediation. Indeed, based on a result where M is fully mediated, we may make the risky conclusion that we know everything we need to know about the process under study and that there is no need to propose a different mechanism. In fact, the finding that some M variables are fully mediated says nothing about the presence or absence of other possible mediators of the effect of X. Moreover, if there is more than one M that fully mediates the effect of X on Y when considered in isolation, it is not very valuable to claim that our preferred mediator does so.

If we explain our full mediation result based on Hayes’ explanations, the fact that the four factors simultaneously included in the model in the effect of self-regulation on the need for psychological help are fully mediated together should not mean that there are no other mediators in this relationship. The full mediation result in the current study does not clearly indicate whether there are different mediators in the effect of self-regulation on the need for psychological help, so it should be kept in mind that there may be different variables that fully or partially mediate the relationship.

## 5. Conclusions

In this study, we present that self-regulation is an effective subjective resource in reducing adolescents’ need for psychological help. Taking the results one step further, we emphasize that mental health professionals should consider self-regulation and related self-efficacy, happiness, problem-solving, and psychological adjustment in interventions aimed at reducing adolescents’ need for help. The contribution of our study to the literature can be expressed as supporting the results of the few studies reporting the effect of self-regulation on the need for psychological help and, more importantly, broadening and better understanding the variables through which this effect occurs. Our study, which examined various mediators in the relationship between self-regulation and need for psychological help, has several limitations. The limitations of the study include the fact that the study group consisted of 1013 adolescents in only one city in Turkey, the inability to obtain results free from random error due to the cross-sectional correlational survey model, the inability to make unambiguous causal inferences, and the collection of data using self-report measures. Finally, the inclusion of the five variables examined in the effect of self-regulation on the need for psychological help as simple mediators in the current study can be considered as a limitation. Because in this effect, the predictive relationships between the existing mediators and the moderators of this effect can be questioned. In this situation, different structural models can be tested in future studies, taking into account the predictive relationships between the existing variables. Additionally, another suggestion is to improve the model by identifying the factors that moderate this effect of self-regulation.

## Figures and Tables

**Table 1:** Descriptive statistics, distributions, and correlations of measurements.

	Range	$\bar{X}$	SD	Skewness	Kurtosis	1	2	3	4	5	6	7	8
1. SRS	21–55	40.77	5.89	−0.181	0.170	1							
2. OHQ	7–35	22.21	5.21	−0.229	−0.276	0.409 ***	1						
3. BRS	6–30	18.13	5.07	−0.059	−0.125	0.247 ***	0.457 ***	1					
4. CSCY	7–28	19.29	3.88	−0.017	−0.081	0.635 ***	0.356 ***	0.226 ***	1				
5. GES	10–40	26.83	5.30	0.138	−0.086	0.620 ***	0.410 ***	0.370 ***	0.632 ***	1			
6. BASE-6	6–42	25.69	9.62	−0.192	−0.864	0.166 ***	0.505 ***	0.503 ***	0.157 ***	0.200 ***	1		
7. PHNS	17–81	40.68	12.96	0.500	−0.018	−0.385 ***	−0.618 ***	−0.354 ***	−0.370 ***	−0.404 ***	−0.449 ***	1	
8. Age	12–19	15.04	1.75	0.092	−1.117	0.024	−0.188 ***	−0.081 **	0.023	−0.091 **	−0.147 ***	0.060	1
9. Grade	7–12	-	-	0.101	−1.359	0.006	−0.216 ***	−0.101 ***	0.013	−0.126 ***	−0.149 ***	0.080 *	0.937 ***

SRS: Self-regulation, OHQ: Happiness, BRS: Psychological resilience, CSCY: Problem solving, GES: Self-efficacy, BASE-6: Psychological adjustment, PHNS: Need for psychological help, *** *p* < 0.001, ** *p* < 0.01, * *p* < 0.05.

## Data Availability

Data from the study are available at https://doi.org/10.6084/m9.figshare.28024043 (accessed on 13 December 2024).
